# Differences in healthcare utilisation between users and non-users of homeopathic products in Spain: Results from three waves of the National Health Survey (2011-2017)

**DOI:** 10.1371/journal.pone.0216707

**Published:** 2019-05-13

**Authors:** Jaime Pinilla, Alejandro Rodriguez-Caro

**Affiliations:** 1 Department of Quantitative Methods, University of Las Palmas de Gran Canaria, Las Palmas de Gran Canaria, Spain; 2 Centre for Research in Health and Economics, Universitat Pompeu Fabra, Barcelona, Spain; National Center for Global Health and Medicine, JAPAN

## Abstract

**Objective:**

To compare the differences in the use of healthcare services: visits to the doctor and hospitalisation, performance of routine tests, and preventive influenza vaccination, between users and non-users of homeopathic products.

**Methods:**

We used the microdata for adults over 15 years old from three waves of the Spanish National Health Survey, corresponding to the years 2011, 2014 and 2017. We proposed a comparative design of a quasi-experimental type, considering as the treatment group the respondents who said that they had used homeopathic products in the past two weeks; and another group, for control, comprising respondents who said that they had not used this type of products, but only conventional medicines, with observable characteristics similar to those of the treatment group. We used a model for rare events logistics regression (relogit) to estimate the probability of using homeopathy. From the propensity score and a vector of control variables, we used techniques of genetic matching to match individuals from the treatment group with similar individuals belonging to the control group.

**Results:**

There are no statistically significant differences between users and non-users of homeopathy in visits to the general practitioner (P>|z| 0.387), to the specialist (P>|z| 0.52), in hospitalisations (P>|z| 0.592) or in the use of emergency services (P>|z| 0.109). Nor were there any statistically significant differences in the performance of routine tests, except for the faecal occult blood test, which is more prevalent in users of homeopathic products. 20.9% of users of homeopathy had done this test compared with 15.3% of non-users (P>|z| 0.022). There are also significant differences in vaccination against influenza with 12.6% of homeopathy users stating that they had been vaccinated in the last influenza campaign, against 21.0% of non-users (P>|z| <0.001). The health conditions which homeopathy users reported were constipation (OR: 1.65 CI: 1.16–2.36), malignant tumour (OR: 1.60 CI: 1.09–2.36) osteoporosis (OR: 1.49 CI: 1.05–2.10), varicose veins (OR: 1.35 CI: 1.05–1.74) and allergy (OR: 1.35 CI: 1.06–1.72).

**Conclusions:**

Differences in the use of healthcare resources between users and non-users of homeopathic products have not been found to be statistically significant in Spain. It has been shown that most homeopathic products are used as a complement to treatment with conventional medicine. Nevertheless, our results highlight some warning signs which should raise the attention of healthcare authorities. The use of these therapies in patients with malignant tumours and the rejection of vaccines are warning signs of a possible health hazard in the long term.

## Introduction

Homeopathy is a therapeutic approach to health problems which does not have the necessary scientific evidence to support either its validity or its usefulness [1,2]. Nevertheless, although it is in regression in some countries [3,4], homeopathy remains popular not only among the general population, but also among healthcare professionals [5–7].

Although its therapeutic efficacy has not been scientifically proved, homeopathy is an option which is sought in many countries. According to data from the European Social Survey of 2014 [8], 5.6% of Europeans said that they had used homeopathy during the past 12 months, a proportion which varies between 13.4% of French and 11.6% of Germans (who use it the most), 2.8% of Spanish, and 1.5% and 1.1% of British and Nordics, respectively.

In Europe, the regulation of the commercialization of homeopathic products, of the diagnostic exercise and of prescription depends on each Member State. In some countries the practice of homeopathy is included, with some considerations, in the coverage of the national healthcare system; in others the healthcare authorities have doubts about its effectiveness. Since 2017, the National Health Service (NHS) in England has recommended that its doctors stop prescribing homeopathy, the reason being the lack of evidence to support its use [9]. According to the House of Commons Science and Technology Committee (HCSTC) in England, homeopathy should not be funded by the NHS, and the Medicines and Healthcare products Regulatory Agency should withdraw recognition of homeopathic products as medicines. The differences in regulation and in the recommendations to healthcare professionals could explain the different proportions of users of homeopathy in each Member State [4].

In Spain, there is no specific regulation of the practice of homeopathy. But the regulation of homeopathic products has been attempted, although in a very confused way, with transitory laws which never become permanent. Homeopathy is practised mainly in private consultations which are advertised as such, or together with other therapies, called “alternative”, under the common denomination of “natural medicine”. The Spanish College of Medicine [*Organización Médica Colegial* (OMC)], the body which represents all the Official Medical Colleges nationally, pronounced itself for the first time about homeopathy in 2011, in a statement in which it recalled that medical professionals are obliged by the standards of the Code of Medical Ethics preferably to use procedures and prescribe drugs whose efficacy has been scientifically proved [10]. Regarding financing by the public healthcare system, homeopathic products and therapies are not financed in Spain.

The Spanish National Health System is characterised by the extension of its benefits to the entire Spanish population, and its objective is to ensure equal access to and use of healthcare services by all citizens, based on their need for assistance. In this sense, one aspect which has not been studied at all is the interaction of the use of alternative therapies with the use of healthcare services, either because of the characteristics of this type of patient and their health problems, or because it could facilitate or hinder access to these services by other users. So far there has been no study in Spain which analyses the impact of the use of non-conventional therapies, and in particular homeopathy, as one of the most popular, on the use of healthcare services. The objective of this work is to try to fill this gap.

## Materials and methods

The sources of information were the Spanish National Health Survey, corresponding to the years 2011 and 2017, and the data for Spain from the European Health Survey of 2014. The geographical scope of these surveys was the entire Spanish territory, our study population being adults aged 15 years and older and living in family homes. The main purpose of the previous surveys was to obtain data about the state of health, its determinant factors, and the use of healthcare services from the citizens’ perspective. In each survey, between 24,000 and 37,500 homes were investigated, distributed among 2,000 and 2,500 census sections respectively. All surveys were household-based with stratified sampling and three clustering stages (for additional details, see S1 and S2 Tables). The datasets included final weights to account for the sampling design.

In this work we propose a quasi-experimental comparative design. The treatment group comprised survey respondents who said that they had used homeopathic products in the past two weeks, and the control group included those respondents who stated that they had not used this type of product but do use conventional medicines. Many researchers are increasingly using population-based sample surveys to estimate the effects of treatments and exposures on health outcomes [11]. However, the groups compared are often different because of lack of randomization. Subjects with specific characteristics may have been more likely to be exposed than other subjects [12]. In such analyses, selecting the appropriate statistical method is essential to minimize the effect of confounding due to measured covariates, as treated subjects frequently differ from control subjects.

### Statistical analysis

Propensity-score matching was performed to compare outcomes between different subject groups, users of homeopathic products versus users of conventional medicines. Introduced by Rosenbaum and Rubin (1983), propensity score (PS) methods help to adjust for selection bias caused by confounding variables associated with both the exposure and outcome.

A logistic regression model was used to calculate the PS for each respondent, and matching was performed using a genetic matching algorithm. The genetic matching algorithm achieves better balance across the treatment and control groups than the classic propensity score or multivariate matching algorithm based on Mahalanobis distance [13].

The independent variables included in the logistic regression model could be grouped into two types of categories: (1) individual, geographic and socioeconomic characteristics of the home: autonomous community of residence, sex, age, marital status, educational level, and social class (constructed from the occupation and level of studies of the person who contributes the most income to the home), and (2) State of health and determinants of health: self-perceived state of health, smoking, physical exercise in leisure time, and body mass index. The medical diagnosis of chronic illnesses in the past 12 months was also included.

The dependent variable was binary, use (Yes = 1, No = 0) of homeopathic products in the past two weeks. Given the low proportion of 1 in the dependent variable, the traditional logit model could underestimate the probability of occurrence of the event by clearly violating the 50/50 balance, so, as an alternative, we proposed an estimate using the weighted logistic regression called rare events logistics regression (relogit) [14]. As prior information about the fraction of ones in the population, we used a mean relative value of users of homeopathy in Spain of 1.16%, with a range between 0.65% and 1.75%, obtained by the National Statistics Institute by inference from the total population in each survey.

From the estimate of the relogit model we obtained the estimated propensity score of an individual belonging to the treatment group using homeopathy. The propensity score summarised all the relevant information contained in the selected independent variables and helped to match individuals in the treatment group with other identical individuals in the control group who were non-users of homeopathy. For the selection of the sample from the control group, a genetic matching was performed, using the previous propensity score [13]. Genetic matching is a generalisation of the propensity score matching proposed by Rosenbaum and Rubin [15] and which avoids the need to manually and iteratively check the propensity score using a search algorithm to iteratively check and improve covariate balance.

Once an appropriate level of balance of the between-group covariates is achieved, the matched data set is ready for the analysis of between-group differences in healthcare utilisation. As a measure of the use of healthcare resources, we analysed the number of visits to the general practitioner and specialist in the past four weeks, and the number of visits to emergency services and hospital admissions in the past 12 months. We also analysed the performance of routine tests: blood pressure, blood cholesterol level measurement, faecal occult blood test, vaginal cytology and mammography. Finally, preventive vaccination against influenza was also analysed.

The average treatment effect on treated (ATT) measured, on average, the effect of homeopathy use on the utilisation of healthcare resources. Assuming that *T* takes two values: 1 = treatment and 0 = control, *U*_*i1*_ and *U*_*i0*_ represent the use of healthcare resources or the performance of routine tests of the individual *i* in the treatment and control group, respectively. The ATT is calculated as E(U_1_-U_0_| T = 1).

The datasets used in this study involved survey designs, and as our objective was to understand population-level effects, sampling weights had to be incorporated in the estimates. As Ridgeway et al. suggest, we used sampling weights in the PS estimation model, relogit model, and in the outcome models, using in these models a final weight as the product of the sampling weight and the propensity score weight [16]. The Population ATT (denoted PATT) is the corresponding ATT estimation for the survey’s target population, accounting for the sampling design. The estimated PATT effect is generated from a weighted regression (Poisson regressions to analyse the healthcare utilization, and logit regression to analyse the performance of routine tests) that incorporates a composite weight which includes the complex survey design.

Statistical analysis was performed using the R packages “Zelig” version 5.1.6 [17] and “Matching” version 4.9–3 [18]. To show the results, we calculated the odds ratios (ORs) and the confidence intervals (CIs) at a 95% confidence level.

## Results

Table 1 shows the percentage distribution of responses about drug use, including homeopathic products, throughout the surveys analysed. The observations in columns T1+T2 relate to the treatment group (665; 1.06%), while column C acts as a control group (40,565; 64.55%).

**Table 1:** Variables which define the treatment group and the control group.

	Drug use in the past two weeks			No drug use in the past two weeks	Don’t Know/No Answer
	T1. Use only homeopathy products	C. Use only conventional medicines	T2. Use both		
2011	7	13,279	224	7,493	4
2014	71	13,452	209	9,089	21
2017	31	13,834	123	9,093	8

Treatment group = T1+T2 Control group = C.

The final number of observations used was 39,855 due to missing values in the independent variables, 7.97% in the treatment group and 3.26% in the control group, respectively. The results of the relogit regression are shown in Table 2. According to these results, the socioeconomic profile of the user of homeopathy is a woman (OR: 2.22; CI: 1.74–2.82), with higher education (OR: 3.63 CI: 2.10–6.30, reference no studies finished), belonging to a high- or middle-class home (professional-managerial class); belonging to a low-class home (unskilled class) carries a 68% lower probability of using homeopathic products than in homes of high social class (OR: 0.32 CI: 0.20–0.50).

**Table 2:** Factors associated with the use of homeopathy in the past two weeks. Estimation by rare event logistic regression incorporating the sampling design weights.

Variable		OR (95% CI)	p-value	Variable	OR (95% CI)	p-value
Gender	Male	1		Education		
	Female	2.22 (1.74–2.82)	<0.01	No studies finished	1	
Age (yr)	15–24	1		Primary	1.41 (0.80–2.46)	0.23
	25–34	0.71 (0.41–1.23)	0.22	Secondary	2.15 (1.29–3.60)	<0.01
	35–44	1.60 (0.96–2.68)	0.07	Post-secondary	2.67 (1.54–4.62)	<0.01
	45–54	1.37 (0.80–2.36)	0.25	First stage tertiary	4.14 (2.44–7.02)	<0.01
	55–64	1.23 (0.68–2.23)	0.48	Second stage tertiary	3.63 (2.10–6.30)	<0.01
	65–74	0.72 (0.38–1.37)	0.32	Social Class		
	+75	0.81 (0.40–1.67)	0.58	Professional occupat.	1	
Civil status	Single	1		Managerial and tech.	1.04 (0.74–1.46)	0.81
	Married	0.82 (0.63–1.06)	0.13	Skilled (non-manual)	0.76 (0.56–1.02)	0.07
	Widowed	1.11 (0.70–1.75)	0.67	Skilled (manual)	0.44 (0.30–0.65)	<0.01
	Divorced	0.96 (0.67–1.39)	0.84	Partly-skilled	0.49 (0.35–0.70)	<0.01
Self-perceived health status				Unskilled occupat.	0.32 (0.20–0.50)	<0.01
	Very good	1		Physical activity		
	Good	1.03 (0.76–1.40)	0.85	None	1	
	Fair	1.18 (0.82–1.70)	0.37	Occasional	1.51 (1.18–1.94)	<0.01
	Bad	1.78 (1.09–2.90)	0.02	Days a month	1.75 (1.27–2.40)	<0.01
	Very bad	1.32 (0.63–2.78)	0.46	Days a week	1.76 (1.27–2.45)	<0.01
Diseases/condition No		1		Asthma	1.20 (0.85–1.69)	0.30
High blood pressure		0.71 (0.54–0.94)	0.02	Constipation	1.65 (1.16–2.36)	0.01
Diabetes		0.80 (0.50–1.30)	0.38	Chronic depression	1.13 (0.83–1.55)	0.43
Varicose veins		1.35 (1.05–1.74)	0.02	Malignant tumour	1.60 (1.09–2.36)	0.02
Neck disorder		1.26 (0.99–1.60)	0.06	Osteoporosis	1.49 (1.05–2.10)	0.03
Allergy		1.35 (1.06–1.72)	0.02	Thyroid	1.32 (0.99–1.77)	0.06
Year (Survey 2011)		1				
Survey 2014		0.92 (0.73–1.16)	0.50			
Survey 2017		0.57 (0.44–0.74)	<0.01			

No obs. = 39,855

Pseudo R^2^ (McFadden) = 0.12

Likelihood ratio test Pr(>Chi-square) <0.000.

Region of residence coefficients are not presented in the table due to space limitations

Regarding the health variables, a user of homeopathy, despite exercising regularly, states that their perceived health is bad, and the OR for homeopathy use among those with bad health (compared to reference: very good health) is 1.78 (CI: 1.06–1.72). The health conditions which homeopathy users reported were constipation (OR: 1.65 CI: 1.16–2.36), malignant tumour (OR: 1.60 CI: 1.09–2.36) osteoporosis (OR: 1.49 CI: 1.05–2.10), varicose veins (OR: 1.35 CI: 1.05–1.74) and allergy (OR: 1.35 CI: 1.06–1.72).

The model also incorporates a categorical variable for the year when the survey was conducted; the significance of the coefficient for 2017 indicates a significant drop in its use of homeopathic products in that year (OR: 0.57 CI: 0.44–0.74, reference survey 2011). Regarding the region of residence, some significant results were obtained, but they are not included in the table due to their low relevance for the analysis.

**Fig 1:**
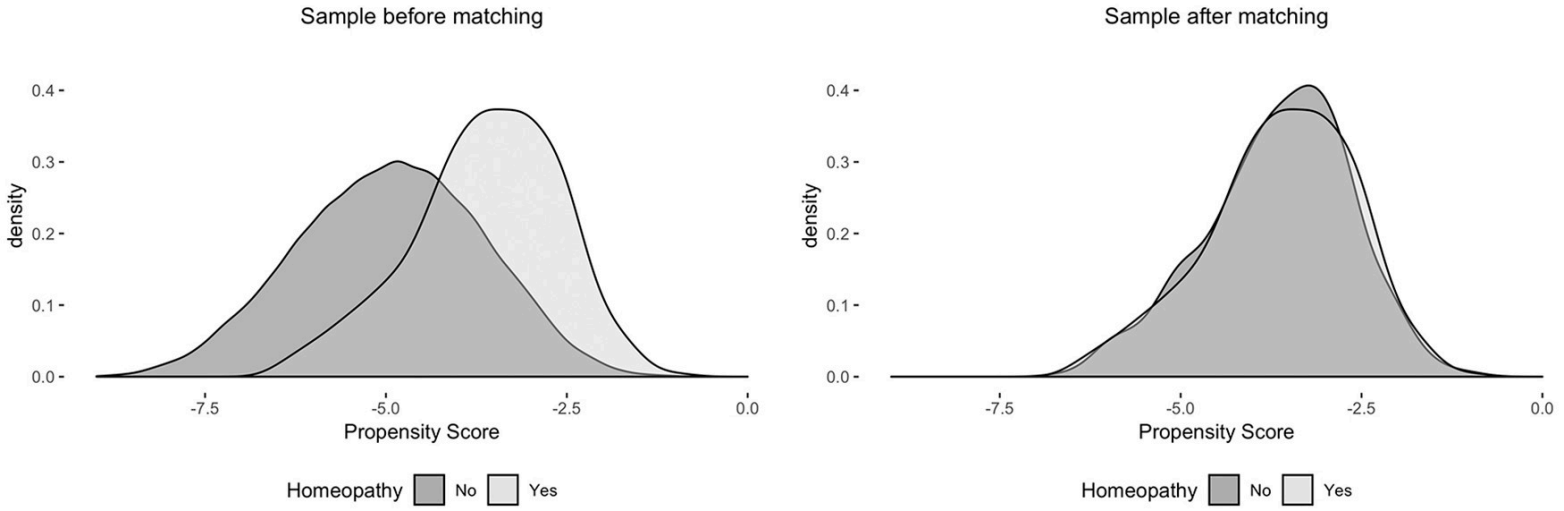
Density functions of the estimated propensity score.

Fig 1 shows the precision achieved in the matching. For this, we compared the density functions of the propensity score for the two groups, treatment and control, before and after the matching. The descriptive analysis of the selected independent variables, before and after the matching, is presented in S3 Table.

The average number of visits to the doctor and hospitalisations for users and non-users of homeopathy, as well as the proportion who attend routine tests or are vaccinated in the influenza campaign are shown in Table 3. It is noted that the average number of visits is very similar for users and non-users of homeopathic products, with a slight difference in favour of users of homeopathy in terms of emergency services, an average of 0.66 versus 0.53. In routine tests, there is also some similarity between treatment and control, except in the faecal occult blood test, in which users of homeopathy outnumber non-users by more than 5 points, 20.9% versus 15.3%, and in preventive vaccination against influenza, in which the relationship is reversed, leaving homeopathy users about 8 points below non-users, 12.6% versus 21.0%.

**Table 3:** Differences in the use of healthcare resources between users and non-users of homeopathy.

Visits to the doctor and hospitalisations							
	Uses of homeopathy products				Uses of homeopathy products		
	Yes	No	No		Yes	No	No
	After matching		Before matching		After matching		Before matching
(%) and means	N = 609	N = 609	N = 38,984	(%) and means	N = 609	N = 609	N = 38,984
General practitioner				Medical specialist			
0	(66.8)	(63.1)	(60.8)	0	(74.2)	(75.4)	(81.2)
1	(25.1)	(28.1)	(31.1)	1	(19.0)	(18.1)	(14.7)
2	(5.9)	(4.4)	(5.4)	2	(4.6)	(4.3)	(2.6)
3	(1.5)	(2.0)	(1.3)	3	(0.3)	(1.3)	(0.8)
≥ 4	(0.7)	(2.5)	(1.4)	≥ 4	(1.8)	(1.0)	(0.7)
Average number of visits	0.44	0.54	0.52	Average number of visits	0.37	0.38	0.26
Emergency services	N = 609	N = 609	N = 38,984	Hospitalisations	N = 609	N = 609	N = 38,984
0	(66.3)	(69.3)	(68.4)	0	(89.5)	(92.9)	(89.3)
1	(21.7)	(20.5)	(20.0)	1	(8.2)	(5.4)	(8.4)
2	(5.7)	(4.4)	(6.4)	2	(2.0)	(0.8)	(1.6)
3	(3.1)	(3.6)	(2.5)	3	(0.3)	(0.3)	(0.4)
≥ 4	(3.1)	(2.1)	(2.7)	≥ 4	(0.0)	(0.3)	(0.4)
Average number of visits	0.66	0.53	0.57	Average number of visits	0.13	0.12	0.15
Routine tests		(%) Yes		(%) Yes	N = 459	N = 459	N = 21,882
Blood cholesterol	(95.6)	(97.4)	(96.1)	Cytology	(91.7)	(88.9)	(76.9)
Blood pressure	(97.2)	(98.2)	(97.6)	Mammography	(73.6)	(74.5)	(67.6)
Faecal occult blood test	(20.9)	(15.3)	(16.6)				
Preventive vaccination Influenza	(12.6)	(21.0)	(28.9)				

Table 4 shows the results of the statistical tests of differences between the treatment group and the control group, under the null hypothesis that there are no differences between the two groups. In the visits to the doctor and hospitalisations, the results do not show statistically significant differences, at 5% level of significance, between users and non-users of homeopathy: P>|z| 0.387 for visits to the general practitioner, P>|z| 0.520 for the specialist doctor, P>|z| 0.592 for hospitalisations and P>|z| 0.109 for visits to emergency services.

**Table 4:** Statistical contrasts in the effect of homeopathy use on the utilisation of healthcare resources. Poisson regression and logistic regression, where the models incorporated the sampling design weights.

Visits to the doctor and hospitalisations	Observ.	*PATT*	Poisson regression P>|z|
Visits to Generalist (in the past 4 weeks)	609	-0.135	0.387
Visits to Specialist (in the past 4 weeks)	609	0.067	0.520
Visits to emergency services (in the past 12 months)	609	-0.045	0.592
Hospitalizations (in the past 12 months)	609	0.108	0.109
Routine tests (Yes, No)			Logistic regression P>|z|
Cytology	459	0.029	0.652
Mammography	459	-0.009	0.878
Blood cholesterol	609	-0.008	0.748
Blood pressure	609	-0.023	0.862
Faecal occult blood test	609	0.042	0.022
Vaccination against influenza	609	-0.094	<0.001

There are no statistically significant differences in the performance of routine tests, except for the faecal occult blood test, which is more prevalent in consumers of homeopathic products, P*ATT* = 0.042 (P>|z| 0.022). Significant differences are shown in relation to influenza vaccination in the last campaign, in this case with negative *PATT* for users of homeopathy, -0.094, and P>|z| <0.001.

## Discussion

In the European Union, homeopathy is the second most widely used modality in alternative medicine after herbal medicine [19]. The key factors in the demand for it range from the construction of a social identity as a user of healthcare products [20] to the balance between perceived benefit and risk [21,22], or the search for more personalised care in response to dissatisfaction or bad experience with conventional medicine [23].

Our results for the Spanish population relate certain socioeconomic characteristics to the use of homeopathic products: being a woman, high educational level, and high social status. This user profile is shared with other developed countries, European [4,24,25] and non-European [3]. According to our results, homeopathy users are positively associated with suffering from constipation, malignant tumour, osteoporosis, varicose veins and allergy, while individuals with a high blood pressure are less likely to use homeopathy. It would be very useful to have details about which conditions were treated with homeopathic products, but the questionnaires of the surveys used in this study did not allow for information about this topic.

Regarding the use of healthcare resources, the results of our study show that users of homeopathic products have resource utilisation, frequency of consultation with the general practitioner, consultations with the specialist doctor, visits to emergency departments and days of hospitalisation which are not statistically different from those of non-users of this type of treatment. Although, a priori, this is contradictory because these products are considered as being within what is called alternative medicine, most homeopathic products are used as a complement to treatments with conventional medicines [26].

In September 2017, the European Academies’ Science Advisory Council (EASAC) summarized, in a 12-page report, some extensive scientific research which concluded that homeopathy is scientifically implausible and produces nothing more than a placebo effect in patients. The EASAC stated that homeopathic remedies can be dangerous because they may delay patients from receiving conventional medical treatment that they need. The EASAC recommended that the European Union member states set up regulations to reject misleading claims and advertisements by homeopaths, remove homeopathic treatments from public health provision, and require that homeopathic product labels clearly describe ingredients and their specific amounts [27].

Although policy recommendations are beyond the scope of this study, the fact that individuals who state that they have been diagnosed with certain chronic problems have a greater predisposition to consume homeopathic products should warrant the attention of those responsible for healthcare policy, particularly in the case of cancer patients, because suffering a tumour is statistically significant in our model of factors which predict the use of homeopathy. Recent studies have found that users of alternative medicines tend to reject conventional treatments, considerably increasing their risk of death [28]. We should also be concerned about the lower demand for vaccines in the influenza campaign from users of homeopathy. Among users of alternative therapies, distrust of vaccines has considerable support, which is leading to a major health problem because they refuse to vaccinate themselves and their children [29].

This paper uses the PS method to address the selection bias in observational studies. Regression models (RM) are the standard tool to appraise multivariate predictors of categorical events and to evaluate the independent predictive role of one or more independent variables of interest. But because of lack of randomisation in the observational studies, subjects with specific characteristics may have been more likely to be exposed than other subjects. If these characteristics (confounders) also affect the outcome, a direct comparison of the groups is likely to produce biased conclusions that may reflect the lack of initial comparability. The simulations indicate that impact estimates based on full unmatched samples are generally more biased, and less robust to miss-specification of the regression function, than those based on matched samples [30]. However, PS models typically result in a smaller sample size which can lead to reduced power. In our study, results were robust to changing the method used, except for the RM for the cytology test, which shows a significant result for homeopathy users. The explanation for this difference could be related to the fact that the PS model works with men and women simultaneously while the RM, in the case of demand for a cytology test, uses only the sample of women. The RM results for all healthcare services are presented in the S4 Table.

Our study has some limitations. First, there is a potential misclassification of users. Since the surveys used in our study rely on self-reported answers, the responses are dependent on respondents’ recall, as well as their willingness to report accurate facts. Nevertheless, we consider that the possible recall bias in our dependent variable, which is use of medicines or homeopathic products, is very low due the relatively short timeframe to which the question refers ("used in the past two weeks"). Secondly, although we focused on the use of homeopathic products, the great diversity of complementary and alternative medicine treatments and their high correlation with the use of homeopathy means that the use of these other products should also be investigated. Unfortunately, the surveys used in this study do not provide this information, so it cannot be analysed here. Finally, our analyses are limited in their causal interpretations because of the cross-sectional design of the surveys. So, we can only observe associations and cannot infer causality. In terms of endogeneity, the following cannot be discarded; omitted variable bias (although given the large amount of baseline variables available from the surveys from which to identify possible covariates for the PS method, we consider that the potential for this bias is low), measurement error, and simultaneity or reversed causality. The causal interpretation of PS matching results rests on the unverifiable assumption that unobserved variables are not correlated with healthcare utilization or with the probability of using homeopathic products. In this regard, the PS method is no panacea for causal research. Replication of this technique on additional appropriate datasets would be an important next step that should be investigated further.

## Conclusion

Differences in the use of healthcare resources between users and non-users of homeopathic products have not been found to be statistically significant in Spain. It has been shown that most homeopathic products are used as a complement to treatment with conventional medicine. Nevertheless, our results highlight some warning signs which should raise the attention of healthcare authorities. The use of these therapies in patients with malignant tumours and the rejection of vaccines are warning signs of a possible health hazard in the long term.

## Supporting information

S1 Table Survey characteristics and sampling design. (DOCX)

S2 Table Baseline characteristics of the variables included in the study. (DOCX)

S3 Table Descriptive analysis of the selected independent variables, before and after the matching. (DOCX)

S4 Table Poisson regressions to analyse the health care utilization. ORs and CI (95%).
(DOCX)

## References

[1] House of Commons Science and Technology Committee. Evidence Check 2 : Homeopathy. Fourth Report of Session 2009–10. In: The Stationery Office Ltd. 2010. http://www.publications.parliament.uk/pa/cm200910/cmselect/cmsctech/45/4502.htm

[2] National Health and Medical Research Council—Australia. NHMRC Statement: Statement on Homeopathy In: Nhmrc. 2015 pp. 1–2. https://nhmrc.gov.au/about-us/publications/evidence-effectiveness-homeopathy-treating-health-conditions

[3] Dossett ML, Davis RB, Kaptchuk TJ, Yeh GY. Homeopathy Use by US Adults: Results of a National Survey. Am J Public Health. American Public Health Association; 2016;106: 743–5. 10.2105/AJPH.2015.303025 26890179 PMC4816083

[4] Kemppainen LM, Kemppainen TT, Reippainen JA, Salmenniemi ST, Vuolanto PH. Use of complementary and alternative medicine in Europe: Health-related and sociodemographic determinants. Scand J Public Health. 2018;46: 448–455. 10.1177/1403494817733869 28975853 PMC5989251

[5] Linde K, Alscher A, Friedrichs C, Wagenpfeil S, Karsch-Völk M, Schneider A. Belief in and use of complementary therapies among family physicians, internists and orthopaedists in Germany—cross-sectional survey. Fam Pract. 2015;32: 62–8. 10.1093/fampra/cmu071 25381009

[6] White AR, Resch KL, Ernst E. Complementary medicine: use and attitudes among GPs. Fam Pract. 1997;14: 302–6. http://www.ncbi.nlm.nih.gov/pubmed/9283851 9283851 10.1093/fampra/14.4.302

[7] von Ammon K, Frei-Erb M, Cardini F, Daig U, Dragan S, Hegyi G, et al Complementary and Alternative Medicine Provision in Europe—First Results Approaching Reality in an Unclear Field of Practices. Forschende Komplementärmedizin / Res Complement Med. 2012;19: 37–43. 10.1159/000343129 23883943

[8] European Social Survey | European Social Survey (ESS) [Internet]. http://www.europeansocialsurvey.org/

[9] HS Clinical Commissioners NE. Items which should not be routinely prescribed in primary care: A Consultation on guidance for CCGs—NHS England—Citizen Space. 2017. https://www.engage.england.nhs.uk/consultation/items-routinely-prescribed/

[10] Organización Médica Colegial de España. Codigo de deontologia medica. Guia de etica medica. 2011. https://www.cgcom.es/codigo_deontologico/files/assets/common/downloads/codigo%20de%20etica.pdf

[11] Austin PC, Jembere N, Chiu M. Propensity score matching and complex surveys. Stat Methods Med Res. SAGE PublicationsSage UK: London, England; 2018;27: 1240–1257. 10.1177/0962280216658920 27460539 PMC5843030

[12] Cepeda MS, Boston R, Farrar JT, Strom BL. Comparison of logistic regression versus propensity score when the number of events is low and there are multiple confounders. Am J Epidemiol. 2003;158: 280–7. Available: http://www.ncbi.nlm.nih.gov/pubmed/12882951 12882951 10.1093/aje/kwg115

[13] Diamond A, Sekhon JS. Genetic Matching for Estimating Causal Effects: A General Multivariate Matching Method for Achieving Balance in Observational Studies. Rev Econ Stat. The MIT Press; 2013;95: 932–945. 10.1162/REST_a_00318

[14] King G, Zeng L. Logistic Regression in Rare Events Data. Polit Anal. Cambridge University Press; 2001;9: 137–163. 10.1093/oxfordjournals.pan.a004868

[15] Rosenbaum PR, Rubin DB. The Central Role of the Propensity Score in Observational Studies for Causal Effects. Biometrika. 1983;70: 41 10.2307/2335942

[16] Ridgeway G, Kovalchik SA, Griffin BA, Kabeto MU. Propensity Score Analysis with Survey Weighted Data. J causal inference. NIH Public Access; 2015;3: 237–249. 10.1515/jci-2014-0039 29430383 PMC5802372

[17] Choirat C, Gandrud C, Honaker J, Imai K, King G, Lau O. Rare Events Logistic Regression in: Zelig Everyone’s Statistical Software [Internet]. 2018. http://docs.zeligproject.org/articles/zelig_relogit.html

[18] Sekhon S, Jasjeet M. Package “Matching”: Multivariate and Propensity Score Matching with Balance Optimization [Internet]. 2018. https://cran.r-project.org/web/packages/Matching/Matching.pdf

[19] Eardley S, Bishop FL, Prescott P, Cardini F, Brinkhaus B, Santos-Rey K, et al A Systematic Literature Review of Complementary and Alternative Medicine Prevalence in EU. Complement Med Res. 2012;19: 18–28. 10.1159/000342708 23883941

[20] Stryker S. Identity Theory and Personality Theory: Mutual Relevance. J Pers. 2007;75: 1083–1102. 10.1111/j.1467-6494.2007.00468.x 17995458

[21] Schiffman LG, Kanuk LL. Consumer behavior. 5th ed Englewood Cliffs NJ: Prentice Hall; 1994 http://www.worldcat.org/title/consumer-behavior/oclc/29182396

[22] Lewith GT, Broomfield J, Prescott P. Complementary cancer care in Southampton: a survey of staff and patients. Complement Ther Med. 2002;10: 100–6. Available: http://www.ncbi.nlm.nih.gov/pubmed/12481958 12481958 10.1054/ctim.2002.0525

[23] Gale N. The Sociology of Traditional, Complementary and Alternative Medicine. Sociol compass. Wiley-Blackwell; 2014;8: 805–822. 10.1111/soc4.12182 25177359 PMC4146620

[24] Menniti-Ippolito F, Gargiulo L, Bologna E, Forcella E, Raschetti R. Use of unconventional medicine in Italy: a nation-wide survey. Eur J Clin Pharmacol. 2002;58: 61–64. 10.1007/s00228-002-0435-8 11956675

[25] Huijts T, Stornes P, Eikemo TA, Bambra C, HiNews Consortium. The social and behavioural determinants of health in Europe: findings from the European Social Survey (2014) special module on the social determinants of health. Eur J Public Health. 2017;27: 55–62. 10.1093/eurpub/ckw231 28355646

[26] MacArtney JI, Wahlberg A. The Problem of Complementary and Alternative Medicine Use Today. Qual Health Res. 2014;24: 114–123. 10.1177/1049732313518977 24406483

[27] European Academies’ Science Advisory Council. Homeopathic products and practices: assessing the evidence and ensuring consistency in regulating medical claims in the EU. http://klnran.ru/2017/02/memorandum02-homeopathy.

[28] Johnson SB, Park HS, Gross CP, Yu JB. Complementary Medicine, Refusal of Conventional Cancer Therapy, and Survival Among Patients With Curable Cancers. JAMA Oncol. 2018;4: 1375 10.1001/jamaoncol.2018.2487 30027204 PMC6233773

[29] Bleser WK, Elewonibi BR, Miranda PY, BeLue R. Complementary and Alternative Medicine and Influenza Vaccine Uptake in US Children. Pediatrics. 2016;138: e20154664 10.1542/peds.2015-4664 27940756 PMC5079075

[30] Rubin DB, Thomas N. Combining Propensity Score Matching with Additional Adjustments for Prognostic Covariates. J Am Stat Assoc. 2000;95: 573 10.2307/2669400

