# Supplementary material for: Differences in healthcare utilisation between users and non-users of homeopathic products in Spain: Results from three waves of the National Health Survey (2011-2017)
Source: PLoS One. 2019 May 13;14(5):e0216707. doi: 10.1371/journal.pone.0216707 (PMC6513046; doi:10.1371/journal.pone.0216707)
Supplement: S1 Table — (DOCX) [file pone.0216707.s001.docx]

**S1 Table. Survey characteristics and sampling design**

| Operation | National Health Survey | European Survey of Health in Spain | National Health Survey |
| --- | --- | --- | --- |
| Objective | To offer the information on the health of the population that is necessary for planning and evaluating health care actions and providing adequate attention to the persons who use the health care services | | |
| Variables studied | Population by sex and age according to: illnesses and accidents suffered, consumption of medication, medical visits, hospitalizations, food habits and consumption of alcohol and tobacco | | |
| Reference period | 2011 | 2014 | 2017 |
| Population scope | People living in family dwellings | Persons aged 15 and over who live in family dwellings. | People living in family dwellings |
| Sample size | 24,000 homes were investigated distributed among 2,000 census sections | 23,000 homes were investigated distributed among 2,500 census sections | 37,500 dwellings distributed in 2,500 census sections |
| Type of sampling | Three-stage stratified sampling | | |
| Collection method | Computer-assisted personal interview | | |
| File in the ISO | 54009 | 54088 | 54009 |
| Source | Spanish National Statistics Institute (Instituto Nacional de Estadística INE, in Spanish) | | |
| Access to microdata | https://www.ine.es/dyngs  /INEbase/en/operacion.htm  ?c=Estadistica_C&cid=12547  36176783&menu=resultad  os&secc=1254736195295  &idp=1254735573175 | https://www.ine.es/dyns  /INEbase/en/operacion.  htm?c=Estadistica_C&cid  =1254736176784&menú  =resultados&secc=12547  36195298&idp=125473  5573175 | https://www.ine.es/dyngs  /INEbase/en/operacion.ht  m?c=Estadistica_C&cid=1  254736176783&menu=re  sultados&secc=12547361  95295&idp=1254735573175 |
| Description of the sampling design | All surveys used in this study were household-based with stratified sampling and three clustering stages; census tracts form the primary sampling units, households are the units of second stage and adults (aged 15 years or older) define the third-stage units; the sample size considered the desired level of precision for the estimates of some indicators at different levels of disaggregation and different population groups (Autonomous Regions); the final weighting was a product of inverse selection probabilities at each stage of the sampling plan, including non-response correction procedures and adjustment calibrations for the known population totals. | | |

Note: ISO (Inventory of Statistical Operations)
