# Supplementary material for: Differences in healthcare utilisation between users and non-users of homeopathic products in Spain: Results from three waves of the National Health Survey (2011-2017)
Source: PLoS One. 2019 May 13;14(5):e0216707. doi: 10.1371/journal.pone.0216707 (PMC6513046; doi:10.1371/journal.pone.0216707)
Supplement: S2 Table — (DOCX) [file pone.0216707.s002.docx]

Table S2 baseline characteristics of the variables included in the study

|  | National Health Survey 2011 | European Survey of Health in Spain 2014 | National Health Survey 2017 |
| --- | --- | --- | --- |
| n | 21007 | 22842 | 23089 |
| Use of medicines (%) |  |  |  |
| No | 7493 (35.7) | 9089 (39.8) | 9093 (39.4) |
| Yes | 13510 (64.3) | 13732 (60.1) | 13988 (60.6) |
| Missing | 4 (0.0) | 21 (0.1) | 8 (0.0) |
| Use of homeopathy (%) |  |  |  |
| No | 20772 (98.9) | 22541 (98.7) | 22927 (99.3) |
| Yes | 231 (1.1) | 280 (1.2) | 154 (0.7) |
| Missing | 4 (0.0) | 21 (0.1) | 8 (0.0) |
| Region (%) |  |  |  |
| Andalucía | 2501 (11.9) | 2606 (11.4) | 2935 (12.7) |
| Aragón | 861 (4.1) | 1052 (4.6) | 1045 (4.5) |
| Asturias | 829 (3.9) | 867 (3.8) | 839 (3.6) |
| Balears | 726 (3.5) | 813 (3.6) | 923 (4.0) |
| Canarias | 1062 (5.1) | 1097 (4.8) | 1120 (4.9) |
| Cantabria | 746 (3.6) | 815 (3.6) | 797 (3.5) |
| Castilla y León | 1299 (6.2) | 1323 (5.8) | 1287 (5.6) |
| Castilla-La Mancha | 1039 (4.9) | 1111 (4.9) | 1127 (4.9) |
| Cataluña | 2270 (10.8) | 2342 (10.3) | 2363 (10.2) |
| Valencia | 1701 (8.1) | 1795 (7.9) | 1831 (7.9) |
| Extremadura | 865 (4.1) | 964 (4.2) | 952 (4.1) |
| Galicia | 1265 (6.0) | 1342 (5.9) | 1335 (5.8) |
| Madrid | 1932 (9.2) | 2451 (10.7) | 2032 (8.8) |
| Murcia | 805 (3.8) | 1013 (4.4) | 1026 (4.4) |
| Navarra | 768 (3.7) | 836 (3.7) | 777 (3.4) |
| País Vasco | 1185 (5.6) | 1264 (5.5) | 1494 (6.5) |
| Rioja | 705 (3.4) | 691 (3.0) | 670 (2.9) |
| Ceuta | 185 (0.9) | 186 (0.8) | 255 (1.1) |
| Melilla | 263 (1.3) | 274 (1.2) | 281 (1.2) |
| Sex = Woman (%) | 11358 (54.1) | 12294 (53.8) | 12494 (54.1) |
| Age (%) |  |  |  |
| 15-24 | 1849 (8.8) | 1773 (7.8) | 1796 (7.8) |
| 25-34 | 2969 (14.1) | 2749 (12.0) | 2421 (10.5) |
| 35-44 | 3934 (18.7) | 4586 (20.1) | 4210 (18.2) |
| 45-54 | 3515 (16.7) | 4052 (17.7) | 4150 (18.0) |
| 55-64 | 3134 (14.9) | 3513 (15.4) | 3823 (16.6) |
| 65-74 | 2710 (12.9) | 2956 (12.9) | 3308 (14.3) |
| +75 | 2896 (13.8) | 3213 (14.1) | 3381 (14.6) |
| CivilStatus (%) |  |  |  |
| Single | 5910 (28.1) | 5960 (26.1) | 5888 (25.5) |
| Married | 10979 (52.3) | 12322 (53.9) | 12465 (54.0) |
| Widowed | 2746 (13.1) | 2939 (12.9) | 2972 (12.9) |
| Divorced | 1351 (6.4) | 1599 (7.0) | 1725 (7.5) |
| Missing | 21 (0.1) | 22 (0.1) | 39 (0.2) |
| Studiess (%) |  |  |  |
| No studies finished | 3069 (14.6) | 2912 (12.7) | 2742 (11.9) |
| Primary | 2683 (12.8) | 5194 (22.7) | 4464 (19.3) |
| Secondary | 6696 (31.9) | 4608 (20.2) | 5531 (24.0) |
| Post-secondary | 2419 (11.5) | 2693 (11.8) | 2866 (12.4) |
| First stage tertiary | 3002 (14.3) | 3175 (13.9) | 3296 (14.3) |
| Second stage tertiary | 3138 (14.9) | 4260 (18.6) | 4190 (18.1) |
| Self-perceived health status (%) |  |  |  |
| Very good | 3736 (17.8) | 4214 (18.4) | 4190 (18.1) |
| Good | 10532 (50.1) | 11174 (48.9) | 11145 (48.3) |
| Fair | 4768 (22.7) | 5191 (22.7) | 5531 (24.0) |
| Bad | 1574 (7.5) | 1678 (7.3) | 1724 (7.5) |
| Very bad | 397 (1.9) | 585 (2.6) | 499 (2.2) |
| High blood pressure (%) |  |  |  |
| No | 15615 (74.3) | 16764 (73.4) | 16843 (72.9) |
| Yes | 5391 (25.7) | 6026 (26.4) | 6244 (27.0) |
| Missing | 1 (0.0) | 52 (0.2) | 2 (0.0) |
| Varicose veins (%) |  |  |  |
| No | 17126 (81.5) | 18959 (83.0) | 20225 (87.6) |
| Yes | 3866 (18.4) | 3867 (16.9) | 2862 (12.4) |
| Missing | 15 (0.1) | 16 (0.1) | 2 (0.0) |
| Neck disorder (%) |  |  |  |
| No | 16643 (79.2) | 18371 (80.4) | 19213 (83.2) |
| Yes | 4351 (20.7) | 4457 (19.5) | 3874 (16.8) |
| Missing | 13 (0.1) | 14 (0.1) | 2 (0.0) |
| Allergy (%) |  |  |  |
| No | 18226 (86.8) | 19256 (84.3) | 19343 (83.8) |
| Yes | 2751 (13.1) | 3560 (15.6) | 3741 (16.2) |
| Missing | 30 (0.1) | 26 (0.1) | 5 (0.0) |
| Asthma (%) |  |  |  |
| No | 19828 (94.4) | 21456 (93.9) | 21707 (94.0) |
| Yes | 1172 (5.6) | 1371 (6.0) | 1381 (6.0) |
| Missing | 7 (0.0) | 15 (0.1) | 1 (0.0) |
| Diabetes (%) |  |  |  |
| No | 19117 (91.0) | 20866 (91.3) | 20823 (90.2) |
| Yes | 1870 (8.9) | 1959 (8.6) | 2266 (9.8) |
| Missing | 20 (0.1) | 17 (0.1) | 0 (0.0) |
| Constipation (%) |  |  |  |
| No | 19778 (94.1) | 21541 (94.3) | 22044 (95.5) |
| Yes | 1222 (5.8) | 1289 (5.6) | 1045 (4.5) |
| Missing | 7 (0.0) | 12 (0.1) | 0 (0.0) |
| Chronic depression (%) |  |  |  |
| No | 18866 (89.8) | 20122 (88.1) | 20621 (89.3) |
| Yes | 2125 (10.1) | 2703 (11.8) | 2464 (10.7) |
| Missing | 16 (0.1) | 17 (0.1) | 4 (0.0) |
| Malignant tumour (%) |  |  |  |
| No | 20230 (96.3) | 21875 (95.8) | 21942 (95.0) |
| Yes | 764 (3.6) | 934 (4.1) | 1146 (5.0) |
| Missing | 13 (0.1) | 33 (0.1) | 1 (0.0) |
| Osteoporosis (%) |  |  |  |
| No | 19732 (93.9) | 21502 (94.1) | 21909 (94.9) |
| Yes | 1198 (5.7) | 1282 (5.6) | 1180 (5.1) |
| Missing | 77 (0.4) | 58 (0.3) | 0 (0.0) |
| Thyroid (%) |  |  |  |
| No | 19716 (93.9) | 21274 (93.1) | 21470 (93.0) |
| Yes | 1256 (6.0) | 1546 (6.8) | 1617 (7.0) |
| Missing | 35 (0.2) | 22 (0.1) | 2 (0.0) |
| Physical activity (%) |  |  |  |
| None | 9390 (44.7) | 8485 (37.1) | 8878 (38.5) |
| Occasional | 7585 (36.1) | 9137 (40.0) | 9042 (39.2) |
| Days a month | 2321 (11.0) | 2542 (11.1) | 2547 (11.0) |
| Days a week | 1695 (8.1) | 2638 (11.5) | 2612 (11.3) |
| Missing | 16 (0.1) | 40 (0.2) | 10 (0.0) |
| Social class (%) |  |  |  |
| ProfesYesonal occupat. | 2143 (10.2) | 2479 (10.9) | 2330 (10.1) |
| Managerial and tech. | 1569 (7.5) | 1881 (8.2) | 1723 (7.5) |
| Skilled (non-manual) | 3785 (18.0) | 4262 (18.7) | 4290 (18.6) |
| Skilled (manual) | 2986 (14.2) | 3285 (14.4) | 3261 (14.1) |
| Partly-skilled | 6711 (31.9) | 7295 (31.9) | 7637 (33.1) |
| Unskilled occupat. | 3052 (14.5) | 3101 (13.6) | 3242 (14.0) |
| Missing | 761 (3.6) | 539 (2.4) | 606 (2.6) |
| Routine test | | | |
| Fecal occult blood (%) |  |  |  |
| No | 18994 (90.4) | 19569 (85.7) | 18564 (80.4) |
| Yes | 1530 (7.3) | 2987 (13.1) | 4352 (18.8) |
| Missing | 483 (2.3) | 286 (1.3) | 173 (0.7) |
| Mammography (%) |  |  |  |
| No | 14278 (68.0) | 15193 (66.5) | 15014 (65.0) |
| Yes | 6662 (31.7) | 7580 (33.2) | 8032 (34.8) |
| Missing | 67 (0.3) | 69 (0.3) | 43 (0.2) |
| Cytology (%) |  |  |  |
| No | 12788 (60.9) | 13662 (59.8) | 13629 (59.0) |
| Yes | 7953 (37.9) | 9005 (39.4) | 9296 (40.3) |
| Missing | 266 (1.3) | 175 (0.8) | 164 (0.7) |
| Blood pressure (%) |  |  |  |
| No | 1460 (7.0) | 905 (4.0) | 661 (2.9) |
| Yes | 19508 (92.9) | 21890 (95.8) | 22402 (97.0) |
| Missing | 39 (0.2) | 47 (0.2) | 26 (0.1) |
| Blood cholesterol (%) |  |  |  |
| No | 2724 (13.0) | 1162 (5.1) | 720 (3.1) |
| Yes | 18141 (86.4) | 21575 (94.5) | 22318 (96.7) |
| Missing | 142 (0.7) | 105 (0.5) | 51 (0.2) |
| Preventive vaccination Influenza = Yes (%) | 4923 (23.4) | 5279 (23.1) | 5327 (23.1) |
| Number of visits to | | | |
| Medical specialist (mean (sd)) | 0.21 (0.66) | 0.20 (0.67) | 0.19 (0.63) |
| General practitioner (mean (sd)) | 0.41 (0.79) | 0.42 (0.82) | 0.40 (0.78) |
| Hospitalisations (mean (sd)) | 0.12 (0.51) | 0.12 (0.50) | 0.12 (0.49) |
| Emergency services (mean (sd)) | 0.45 (1.14) | 0.47 (1.27) | 0.53 (1.30) |
