# Supplementary material for: Differences in healthcare utilisation between users and non-users of homeopathic products in Spain: Results from three waves of the National Health Survey (2011-2017)
Source: PLoS One. 2019 May 13;14(5):e0216707. doi: 10.1371/journal.pone.0216707 (PMC6513046; doi:10.1371/journal.pone.0216707)
Supplement: S3 Table — (DOCX) [file pone.0216707.s003.docx]

**S3 Table. Descriptive analysis of the selected independent variables, before and after the matching**

| Before matching | | | | After matching | | |
| --- | --- | --- | --- | --- | --- | --- |
|  | Homeopathy | | (p-value) | Homeopathy | | (p-value) |
| Variable | Yes | No |  | Yes | No |  |
| Nº. observations | 609 | 38,984 |  | 609 | 609 |  |
| Region (%) |  |  | <0.001 |  |  | 0.938 |
| Andalucía | 41 (6.7) | 4,791 (12.3) |  | 41 (6.7) | 39 (6.4) |  |
| Aragón | 47 (7.7) | 1,656 (4.2) |  | 47 (7.7) | 38 (6.2) |  |
| Asturias | 18 (3.0) | 1,557 (4.0) |  | 18 (3.0) | 27 (4.4) |  |
| Balears | 31 (5.1) | 1,162 (3.0) |  | 31 (5.1) | 25 (4.1) |  |
| Canarias | 46 (7.6) | 1,850 (4.7) |  | 46 (7.6) | 37 (6.1) |  |
| Cantabria | 11 (1.8) | 1,108 (2.8) |  | 11 (1.8) | 9 (1.5) |  |
| Castilla y León | 12 (2.0) | 2,398 (6.2) |  | 12 (2.0) | 13 (2.1) |  |
| Castilla-La Mancha | 16 (2.6) | 1,750 (4.5) |  | 16 (2.6) | 23 (3.8) |  |
| Cataluña | 98 (16.1) | 4,033 (10.3) |  | 98 (16.1) | 101 (16.6) |  |
| Valencia | 60 (9.9) | 3,387 (8.7) |  | 60 (9.9) | 67 (11.0) |  |
| Extremadura | 7 (1.1) | 1,605 (4.1) |  | 7 (1.1) | 9 (1.5) |  |
| Galicia | 23 (3.8) | 2,221 (5.7) |  | 23 (3.8) | 23 (3.8) |  |
| Madrid | 74 (12.2) | 3,874 (9.9) |  | 74 (12.2) | 80 (13.1) |  |
| Murcia | 24 (3.9) | 1,702 (4.4) |  | 24 (3.9) | 22 (3.6) |  |
| Navarra | 40 (6.6) | 1,514 (3.9) |  | 40 (6.6) | 33 (5.4) |  |
| País Vasco | 37 (6.1) | 2,353 (6.0) |  | 37 (6.1) | 41 (6.7) |  |
| Rioja | 20 (3.3) | 1,279 (3.3) |  | 20 (3.3) | 20 (3.3) |  |
| Ceuta | 2 (0.3) | 327 (0.8) |  | 2 (0.3) | 2 (0.3) |  |
| Melilla | 2 (0.3) | 417 (1.1) |  | 2 (0.3) | 0 (0.0) |  |
| Year (%) |  |  | <0.001 |  |  | 0.025 |
| 2011 | 208 (34.2) | 10768 (27.6) |  | 208 (34.2) | 174 (28.6) |  |
| 2014 | 249 (40.9) | 14028 (36.0) |  | 249 (40.9) | 295 (48.4) |  |
| 2017 | 152 (25.0) | 14188 (36.4) |  | 152 (25.0) | 140 (23.0) |  |
| Sex = Women (%) | 458 (75.2) | 21843 (56.0) | <0.001 | 458 (75.2) | 457 (75.0) | 1.000 |
| Years (Intervals) (%) |  |  | <0.001 |  |  | 0.817 |
| [15-24] | 28 (4.6) | 2122 (5.4) |  | 28 (4.6) | 25 (4.1) |  |
| [25-34] | 52 (8.5) | 3653 (9.4) |  | 52 (8.5) | 46 (7.6) |  |
| [35-44] | 157 (25.8) | 6295 (16.1) |  | 157 (25.8) | 172 (28.2) |  |
| [45-54] | 161 (26.4) | 6783 (17.4) |  | 161 (26.4) | 154 (25.3) |  |
| [55-64] | 112 (18.4) | 7145 (18.3) |  | 112 (18.4) | 116 (19.0) |  |
| [65-74] | 59 (9.7) | 6648 (17.1) |  | 59 (9.7) | 49 (8.0) |  |
| Age +75 | 40 (6.6) | 6338 (16.3) |  | 40 (6.6) | 47 (7.7) |  |
|  | | | | | | |

**S1 Table. Continuation (Descriptive analysis of the selected independent variables, before and after the matching)**

| Marital Status (%) |  |  | <0.001 |  |  | 0.418 |
| --- | --- | --- | --- | --- | --- | --- |
| Single | 164 (26.9) | 8495 (21.8) |  | 164 (26.9) | 143 (23.5) |  |
| Married | 310 (50.9) | 22080 (56.6) |  | 310 (50.9) | 337 (55.3) |  |
| Widowed | 65 (10.7) | 5539 (14.2) |  | 65 (10.7) | 65 (10.7) |  |
| Divorced | 70 (11.5) | 2870 (7.4) |  | 70 (11.5) | 64 (10.5) |  |
| Highest education degree (%) |  |  | <0.001 |  |  | 0.752 |
| Not finished primary | 26 (4.3) | 5550 (14.2) |  | 26 (4.3) | 26 (4.3) |  |
| Primary | 51 (8.4) | 8082 (20.7) |  | 51 (8.4) | 51 (8.4) |  |
| Secondary | 118 (19.4) | 9321 (23.9) |  | 118 (19.4) | 108 (17.7) |  |
| Post-secondary | 81 (13.3) | 4372 (11.2) |  | 81 (13.3) | 96 (15.8) |  |
| First stage tertiary | 128 (21.0) | 5135 (13.2) |  | 128 (21.0) | 138 (22.7) |  |
| Second stage tertiary | 205 (33.7) | 6524 (16.7) |  | 205 (33.7) | 190 (31.2) |  |
| Self-perceived health status (%) | |  | 0.463 |  |  | 0.121 |
| Very Good | 77 (12.6) | 4476 (11.5) |  | 77 (12.6) | 58 (9.5) |  |
| Good | 288 (47.3) | 18465 (47.4) |  | 288 (47.3) | 330 (54.2) |  |
| Fair | 164 (26.9) | 11359 (29.1) |  | 164 (26.9) | 156 (25.6) |  |
| Bad | 66 (10.8) | 3627 (9.3) |  | 66 (10.8) | 53 (8.7) |  |
| Very Bad | 14 (2.3) | 1057 (2.7) |  | 14 (2.3) | 12 (2.0) |  |
| High blood pressure =Si (%) | 132 (21.7) | 13623 (34.9) | <0.001 | 132 (21.7) | 128 (21.0) | 0.834 |
| Varicose veins =Si (%) | 147 (24.1) | 7198 (18.5) | <0.001 | 147 (24.1) | 145 (23.8) | 0.946 |
| Neck Disorder =Si (%) | 185 (30.4) | 9055 (23.2) | <0.001 | 185 (30.4) | 182 (29.9) | 0.901 |
| Allergy =Si (%) | 166 (27.3) | 6794 (17.4) | <0.001 | 166 (27.3) | 166 (27.3) | 1 |
| Asthma =Si (%) | 64 (10.5) | 2808 (7.2) | 0.002 | 64 (10.5) | 65 (10.7) | 1 |
| Diabetes =Si (%) | 28 (4.6) | 4769 (12.2) | <0.001 | 28 (4.6) | 23 (3.8) | 0.567 |
| Constipation =Si (%) | 66 (10.8) | 2539 (6.5) | <0.001 | 66 (10.8) | 66 (10.8) | 1 |
| Chronic depression =Si (%) | 109 (17.9) | 5457 (14.0) | 0.007 | 109 (17.9) | 99 (16.3) | 0.493 |
| Malignant tumour =Si (%) | 46 (7.6) | 2169 (5.6) | 0.042 | 46 (7.6) | 46 (7.6) | 1 |
| Osteoporosis =Si (%) | 64 (10.5) | 2687 (6.9) | 0.001 | 64 (10.5) | 64 (10.5) | 1 |
| Thyroid =Si (%) | 85 (14.0) | 3513 (9.0) | <0.001 | 85 (14.0) | 85 (14.0) | 1 |
| Physical Activity (%) |  |  | <0.001 |  |  | 0.643 |
| None | 165 (27.1) | 15661 (40.2) |  | 165 (27.1) | 161 (26.4) |  |
| Occasional | 253 (41.5) | 15919 (40.8) |  | 253 (41.5) | 273 (44.8) |  |
| Days a month | 104 (17.1) | 3678 (9.4) |  | 104 (17.1) | 99 (16.3) |  |
| Days a week | 87 (14.3) | 3726 (9.6) |  | 87 (14.3) | 76 (12.5) |  |
| Social Class (%) |  |  | <0.001 |  |  | 0.589 |
| Professional occupat. | 128 (21.0) | 4103 (10.5) |  | 128 (21.0) | 113 (18.6) |  |
| Managerial and tech. | 89 (14.6) | 2998 (7.7) |  | 89 (14.6) | 93 (15.3) |  |
| Skilled (non-manual) | 153 (25.1) | 7389 (19.0) |  | 153 (25.1) | 160 (26.3) |  |
| Skilled (manual) | 61 (10.0) | 5866 (15.0) |  | 61 (10.0) | 71 (11.7) |  |
| Partly-skilled | 137 (22.5) | 13008 (33.4) |  | 137 (22.5) | 142 (23.3) |  |
| Unskilled occupat. | 41 (6.7) | 5620 (14.4) |  | 41 (6.7) | 30 (4.9) |  |
