# Supplementary material for: Differences in healthcare utilisation between users and non-users of homeopathic products in Spain: Results from three waves of the National Health Survey (2011-2017)
Source: PLoS One. 2019 May 13;14(5):e0216707. doi: 10.1371/journal.pone.0216707 (PMC6513046; doi:10.1371/journal.pone.0216707)
Supplement: S4 Table — ORs and CI (95%). (DOCX) [file pone.0216707.s004.docx]

Poisson regressions to analyse the health care utilization. ORs and CI (95%)

|  | Number of visits to | | | |
| --- | --- | --- | --- | --- |
|  | General practitioner | Medical specialist | Hospitalisations | Emergency services |
| Intercept | 0.223  (0.19-0.26) | 0.091  (0.07-0.12) | 0.042  (0.03-0.06) | 0.589  (0.49-0.71) |
| Use of Homeopathy (reference = No) | |  |  |  |
| Yes | 0.89  (0.76-1.04) | 1.124  (0.93-1.36) | 0.962  (0.7-1.33) | 0.97  (0.81-1.17) |
| Year (reference = Survey 2011) | |  |  |  |
| Survey 2014 | 1.072  (1.02-1.13) | 0.955  (0.87-1.04) | 0.923  (0.82-1.04) | 1.013  (0.94-1.09) |
| Survey 2017 | 1.032  (0.98-1.08) | 0.929  (0.86-1.01) | 0.952  (0.85-1.07) | 1.134  (1.06-1.21) |
| Sex (reference = male) |  |  |  |  |
| Female | 1.068  (1.02-1.12) | 1.07  (1-1.15) | 0.758  (0.69-0.84) | 1.11  (1.04-1.18) |
| Age (reference = 15-24) |  |  |  |  |
| 25-34 | 1.029  (0.91-1.16) | 1.178  (0.99-1.4) | 1.03  (0.76-1.39) | 0.768  (0.67-0.88) |
| 35-44 | 0.953  (0.85-1.07) | 1.076  (0.9-1.29) | 0.9  (0.68-1.19) | 0.563  (0.49-0.65) |
| 45-54 | 0.924  (0.82-1.04) | 0.982  (0.82-1.17) | 0.762  (0.57-1.01) | 0.363  (0.32-0.42) |
| 55-64 | 0.948  (0.84-1.07) | 0.895  (0.75-1.07) | 0.773  (0.58-1.03) | 0.283  (0.24-0.33) |
| 65-74 | 0.995  (0.88-1.13) | 0.903  (0.75-1.09) | 0.948  (0.7-1.28) | 0.249  (0.21-0.29) |
| 75 | 1.06  (0.93-1.21) | 0.794  (0.63-0.99) | 0.99  (0.74-1.33) | 0.274  (0.23-0.33) |
| Civil Status (reference = single) | |  |  |  |
| Married | 1.081  (1.02-1.15) | 1.113  (1-1.23) | 1.161  (1.02-1.33) | 1.106  (1.01-1.21) |
| Widowed | 1.006  (0.92-1.1) | 0.942  (0.82-1.09) | 1.192  (0.98-1.45) | 1.045  (0.93-1.18) |
| Divorced | 1.1  (1.01-1.2) | 1.12  (0.97-1.29) | 1.34  (1.09-1.64) | 1.345  (1.19-1.52) |
| Studies (reference = No studies finished) | | |  |  |
| Primary | 0.97  (0.91-1.03) | 1.068  (0.95-1.2) | 0.974  (0.85-1.12) | 0.848  (0.77-0.94) |
| Secondary | 1.038  (0.97-1.11) | 1.166  (1.02-1.33) | 1.048  (0.9-1.22) | 0.876  (0.79-0.98) |
| Post-secondary | 1.075  (0.98-1.17) | 1.432  (1.23-1.67) | 1.086  (0.91-1.29) | 0.848  (0.75-0.96) |
| First stage tertiary | 1.048  (0.96-1.14) | 1.408  (1.22-1.62) | 1.196  (0.99-1.44) | 0.873  (0.77-0.98) |
| Second stage tertiary | 0.983  (0.9-1.08) | 1.741  (1.49-2.04) | 1.326  (1.09-1.61) | 0.844  (0.75-0.96) |
| Self-perceived health status (reference = Very good) | | |  |  |
| Good | 1.246  (1.15-1.35) | 1.246  (1.09-1.42) | 1.493  (1.21-1.84) | 1.338  (1.21-1.48) |
| Fair | 1.981  (1.82-2.16) | 2.669  (2.31-3.08) | 4.159  (3.36-5.15) | 2.805  (2.5-3.14) |
| Bad | 2.772  (2.51-3.06) | 4.352  (3.71-5.11) | 9.892  (7.85-12.46) | 4.467 (3.93-5.08) |
| Very bad | 3.264  (2.85-3.74) | 6.308  (4.94-8.05) | 15.805  (11.89-21.01) | 6.425  (5.41-7.63) |
| Diseases/condition (reference = no) | |  |  |  |
| High blood pressure | 1.127  (1.08-1.18) | 0.99  (0.92-1.07) | 0.996  (0.9-1.1) | 1.135  (1.06-1.22) |
| Varicose veins | 1.014  (0.97-1.06) | 1.01  (0.93-1.1) | 1.053  (0.94-1.18) | 1.139  (1.05-1.23) |
| Neck disorder | 1.09  (1.04-1.14) | 1.044 (0.97-1.13) | 0.872  (0.78-0.97) | 1.121  (1.05-1.2) |
| Allergy | 1.066  (1.01-1.12) | 1.008  (0.93-1.09) | 0.987  (0.88-1.11) | 1.144  (1.06-1.23) |
| Asthma | 1.174  (1.09-1.26) | 1.098  (0.98-1.23) | 1.215  (1.04-1.41) | 1.21  (1.1-1.33) |
| Diabetes | 1.106  (1.05-1.17) | 1.088  (0.97-1.22) | 1.182  (1.05-1.33) | 1.083  (1-1.17) |
| Constipation | 1.129  (1.05-1.22) | 1.168  (1.05-1.3) | 1.13  (0.93-1.37) | 1.102  (1-1.22) |
| Chronic depression | 1.141  (1.08-1.2) | 1.183  (1.08-1.29) | 1.044  (0.93-1.18) | 1.161  (1.07-1.26) |
| Malignant tumour | 1.098  (1.02-1.18) | 1.774  (1.6-1.96) | 1.933  (1.68-2.23) | 1.195  (1.07-1.33) |
| Osteoporosis | 1.032  (0.95-1.12) | 1.102  (0.98-1.24) | 0.968  (0.83-1.12) | 1.102  (0.98-1.24) |
| Thyroid | 1.043  (0.98-1.11) | 1.068  (0.98-1.17) | 0.978  (0.85-1.13) | 1.001  (0.91-1.11) |
| Physical activity (reference = none) | |  |  |  |
| Occasional | 0.983  (0.94-1.03) | 0.928  (0.86-1.01) | 0.787  (0.71-0.87) | 0.906  (0.85-0.96) |
| Days a month | 1.008  (0.93-1.09) | 0.921  (0.82-1.03) | 0.659  (0.56-0.78) | 0.945  (0.85-1.05) |
| Days a week | 0.882  (0.82-0.95) | 0.925  (0.82-1.04) | 0.655  (0.55-0.78) | 0.948  (0.85-1.06) |
| Social class (reference = Professional occupat.) | | |  |  |
| Managerial and tech. | 1.084  (0.98-1.2) | 1.004  (0.87-1.16) | 1.127  (0.9-1.41) | 1.058  (0.94-1.19) |
| Skilled (non-manual) | 1.115  (1.02-1.22) | 0.933  (0.82-1.06) | 1.017  (0.86-1.21) | 1.114  (1-1.24) |
| Skilled (manual) | 1.177  (1.07-1.29) | 0.942  (0.81-1.09) | 1.076  (0.88-1.31) | 1.178  (1.05-1.32) |
| Partly-skilled | 1.15  (1.05-1.26) | 0.798  (0.7-0.9) | 1.042  (0.87-1.24) | 1.144  (1.03-1.27) |
| Unskilled occupat. | 1.16  (1.06-1.28) | 0.811  (0.69-0.95) | 1.023  (0.84-1.24) | 1.143  (1.01-1.29) |

*p<0.1; **p<0.05; ***p<0.01

Logit regression to analyse the performance of routine tests. ORs and CI (95%)

|  | Routine tests | | | | | | | | | | | | | | | | | | | |  |  |  |
| --- | --- | --- | --- | --- | --- | --- | --- | --- | --- | --- | --- | --- | --- | --- | --- | --- | --- | --- | --- | --- | --- | --- | --- |
|  | Blood pressure | | Blood cholesterol | | | Faecal occult blood | | | Mammography | | | Cytology | | | | Preventive vaccination Influenza | | | |  |  |  |  |
| Intercept | 1.439  (0.82-2.53) | | 0.503  (0.32-0.8) | | | 0.012  (0.01-0.02) | | | 0.024  (0.02-0.04) | | | 0.101 (0.07-0.14) | | | | 0.061  (0.04-0.08) | | | |  |  |  |  |
| Use of Homeopathy (reference = No) | | | | | |  | | |  | | |  | | | |  | | | |  |  |  |  |
| Yes | 0.838  (0.47-1.5) | | 0.735  (0.44-1.23) | | | 1.297  (1-1.68) | | | 1.204  (0.88-1.65) | | | 1.751 (1.02-2.99) | | | | 0.589  (0.43-0.81) | | | |  |  |  |  |
| Year (reference = Survey 2011) | | | |  | | |  | | |  | | | |  | | | |  | | | | |  |
| Survey 2014 | 1.413  (1.18-1.7) | | 2.491  (2.13-2.91) | | | 1.925  (1.74-2.13) | | | 1.072  (0.95-1.21) | | | 0.995 (0.88-1.12) | | | | 0.872  (0.8-0.95) | | | |  |  |  |  |
| Survey 2017 | 2.063  (1.68-2.53) | | 4.494  (3.75-5.39) | | | 2.981  (2.71-3.28) | | | 1.16  (1.03-1.3) | | | 1.098 (0.97-1.24) | | | | 0.795  (0.73-0.86) | | | |  |  |  |  |
| Sex (reference = male) | |  | | |  | | |  | | |  | | | |  | | | |  | | | | |
| Female | 1.1  (0.93-1.3) | | 1.088  (0.94-1.26) | | | 0.867  (0.8-0.93) | | |  | | | |  | | | | 0.835  (0.78-0.9) | | | | |  |  |
| Age (reference = 15-24) | | |  | | |  | | |  | | | |  | | | |  | | | | |  |  |
| 25-34 | 2.533  (1.99-3.23) | | 3.3  (2.67-4.08) | | | 1.474  (1.13-1.92) | | | 2.217  (1.62-3.03) | | | | 5.24  (4.22-6.51) | | | | 1.064  (0.8-1.42) | | | | |  |  |
| 35-44 | 2.963  (2.26-3.89) | | 5.147  (4.04-6.55) | | | 1.477  (1.15-1.91) | | | 8.209  (6.08-11.08) | | | | 6.591 (5.24-8.3) | | | | 1.44  (1.1-1.89) | | | | |  |  |
| 45-54 | 3.873 (  2.85-5.27) | | 6.378  (4.83-8.42) | | | 2.614  (2.04-3.35) | | | 68.057  (49.64-93.3) | | | | 6.801 (5.38-8.6) | | | | 2.205  (1.68-2.89) | | | | |  |  |
| 55-64 | 5.336  (3.6-7.9) | | 10.373  (7.41-14.52) | | | 4.164  (3.24-5.35) | | | 300.979  (207.0-437.4) | | | | 4.712 (3.72-5.96) | | | | 4.707  (3.59-6.16) | | | | |  |  |
| 65-74 | 6.563  (4.03-10.7) | | 12.652  (8.66-18.48) | | | 3.828  (2.96-4.95) | | | 215.123  (149.9-308.5) | | | | 2.538  (2-3.22) | | | | 14.36  (10.94-18.85) | | | | |  |  |
| 75 | 15.702 (8.72-28.26) | | 13.444  (9.03-20.01) | | | 2.551  (1.95-3.34) | | | 33.944  (24.02-47.97) | | | | 0.984 (0.77-1.26) | | | | 27.742 (20.97-36.71) | | | | |  |  |
| Civil Status (reference = single) | | |  | | |  | | |  | | | |  | | | |  | | | | |  |  |
| Married | 1.505  (1.2-1.89) | | 1.289  (1.07-1.56) | | | 0.997  (0.9-1.11) | | | 1.726  (1.5-1.98) | | | | 3.053 (2.62-3.56) | | | | 0.937  (0.84-1.05) | | | | |  |  |
| Widowed | 1.37  (0.83-2.27) | | 1.218  (0.85-1.74) | | | 0.924  (0.8-1.07) | | | 1.16  (0.97-1.39) | | | | 2.447 (2.04-2.93) | | | | 0.778  (0.68-0.89) | | | | |  |  |
| Divorced | 1.301  (0.88-1.93) | | 1.191  (0.86-1.65) | | | 1.032  (0.88-1.21) | | | 1.723  (1.38-2.16) | | | | 2.973 (2.27-3.9) | | | | 0.788  (0.66-0.94) | | | | |  |  |
| Studies (reference = No studies finished) | | | | | |  | | |  | | | |  | | | |  | | | | |  |  |
| Primary | 1.016  (0.66-1.56) | | 1.142  (0.83-1.57) | | | 1.188  (1.05-1.34) | | | 1.287  (1.09-1.52) | | | | 1.705 (1.48-1.96) | | | | 0.97  (0.88-1.07) | | | | |  |  |
| Secondary | 0.967  (0.64-1.46) | | 1.318  (0.96-1.81) | | | 1.383  (1.21-1.58) | | | 1.612  (1.32-1.96) | | | | 2.365 (2.01-2.78) | | | | 0.81  (0.72-0.91) | | | | |  |  |
| Post-secondary | 0.991  (0.63-1.56) | | 1.488  (1.04-2.12) | | | 1.486  (1.27-1.74) | | | 1.668  (1.32-2.1) | | | | 3.338 (2.67-4.18) | | | | 0.737  (0.64-0.85) | | | | |  |  |
| First stage tertiary | 1.434  (0.91-2.26) | | 2.295  (1.61-3.28) | | | 1.455  (1.25-1.69) | | | 1.738  (1.39-2.17) | | | | 5.075 (4.05-6.36) | | | | 0.77  (0.66-0.89) | | | | |  |  |
| Second stage tertiary | 1.361  (0.85-2.17) | | 2.571  (1.77-3.74) | | | 1.567  (1.34-1.84) | | | 2.05  (1.63-2.57) | | | | 5.486 (4.33-6.95) | | | | 0.923  (0.79-1.07) | | | | |  |  |
| Self-perceived health status (reference = Very good) | | | | | | | | |  | | | |  | | | |  | | | | |  |  |
| Good | 1.106  (0.91-1.34) | | 1.157  (0.98-1.37) | | | 1.365  (1.19-1.56) | | | 1.068  (0.92-1.24) | | | | 1.19  (1.01-1.4) | | | | 1.216  (1.07-1.39) | | | | |  |  |
| Fair | 1.479  (1.12-1.94) | | 1.512  (1.21-1.89) | | | 1.687  (1.46-1.95) | | | 1.071  (0.9-1.27) | | | | 1.013 (0.85-1.21) | | | | 1.473  (1.28-1.69) | | | | |  |  |
| Bad | 1.822  (1.14-2.91) | | 1.673  (1.17-2.4) | | | 1.81  (1.52-2.15) | | | 0.894  (0.72-1.11) | | | | 0.92  (0.73-1.15) | | | | 1.547  (1.31-1.82) | | | | |  |  |
| Very bad | 1.735  (0.67-4.5) | | 3.197  (1.33-7.67) | | | 1.986  (1.58-2.49) | | | 0.848  (0.62-1.15) | | | | 0.932 (0.69-1.27) | | | | 1.473  (1.17-1.85) | | | | |  |  |
| Diseases/condition (reference = no) | | |  | | |  | | |  | | | |  | | | |  | | | | |  |  |
| High blood pressure | 12.084  (7.21-20.25) | | 1.644  (1.33-2.03) | | | 1.024  (0.95-1.1) | | | 1.02  (0.91-1.15) | | | | 0.927 (0.83-1.04) | | | | 1.218  (1.14-1.3) | | | | |  |  |
| Varicose veins | 0.97  (0.74-1.27) | | 1.042  (0.84-1.29) | | | 1.125  (1.03-1.23) | | | 1.183  (1.06-1.32) | | | | 1.229  (1.1-1.38) | | | | 1.093  (1.01-1.19) | | | | |  |  |
| Neck disorder | 1.281  (1-1.64) | | 0.921  (0.77-1.11) | | | 1.197  (1.1-1.3) | | | 1.284  (1.15-1.44) | | | | 1.265 (1.13-1.41) | | | | 0.96  (0.89-1.04) | | | | |  |  |
| Allergy | 1.382  (1.11-1.71) | | 1.269  (1.05-1.54) | | | 1.217  (1.11-1.33) | | | 1.159  (1.03-1.31) | | | | 1.117 (0.98-1.27) | | | | 1.098  (1-1.2) | | | | |  |  |
| Asthma | 1.059  (0.77-1.46) | | 0.892  (0.68-1.16) | | | 1.069  (0.94-1.22) | | | 0.858  (0.72-1.02) | | | | 0.981 (0.82-1.17) | | | | 2.051  (1.8-2.33) | | | | |  |  |
| Diabetes | 1.491  (0.91-2.45) | | 3.015  (1.95-4.66) | | | 1.172  (1.06-1.29) | | | 0.932  (0.79-1.1) | | | | 0.945 (0.82-1.09) | | | | 1.793  (1.63-1.97) | | | | |  |  |
| Constipation | 1.081  (0.7-1.68) | | 0.926  (0.66-1.3) | | | 1.507  (1.33-1.71) | | | 1.208  (1.02-1.43) | | | | 1.105 (0.94-1.3) | | | | 1.203  (1.07-1.36) | | | | |  |  |
| Chronic depression | 1.047  (0.76-1.43) | | 1.336  (1.01-1.76) | | | 1.222  (1.11-1.35) | | | 1.067  (0.93-1.22) | | | | 1.145  (1-1.31) | | | | 0.976  (0.89-1.07) | | | | |  |  |
| Malignant tumour | 2.523  (1.13-5.62) | | 2.344  (1.28-4.28) | | | 1.605  (1.42-1.82) | | | 2.751  (2.12-3.57) | | | | 2.138 (1.73-2.64) | | | | 0.998  (0.88-1.13) | | | | |  |  |
| Osteoporosis | 0.558  (0.32-0.96) | | 1.182  (0.77-1.81) | | | 1.255  (1.1-1.43) | | | 1.205  (1.01-1.43) | | | | 1.482 (1.28-1.72) | | | | 1.108  (0.98-1.25) | | | | |  |  |
| Thyroid | 1.573  (1.06-2.33) | | 3.109  (2.06-4.7) | | | 1.064  (0.95-1.19) | | | 1.259  (1.1-1.44) | | | | 1.157  (1-1.33) | | | | 1.069  (0.95-1.2) | | | | |  |  |
| Physical activity (reference = none) | | |  | | |  | | |  | | | |  | | | |  | | | | |  |  |
| Occasional | 1.078  (0.89-1.3) | | 1.042  (0.89-1.23) | | | 1.105  (1.02-1.19) | | | 1.155  (1.04-1.28) | | | | 1.197 (1.08-1.33) | | | | 1.164  (1.08-1.25) | | | | |  |  |
| Days a month | 1.124  (0.87-1.45) | | 1.107  (0.89-1.37) | | | 1.218  (1.07-1.39) | | | 1.311  (1.1-1.57) | | | | 1.597  (1.29-1.98) | | | | 1.057  (0.92-1.21) | | | | |  |  |
| Days a week | 1.13  (0.88-1.45) | | 0.908  (0.74-1.12) | | | 1.306  (1.15-1.49) | | | 1.385  (1.16-1.65) | | | | 1.556  (1.29-1.88) | | | | 0.937  (0.82-1.07) | | | | |  |  |
| Social class (reference = Professional occupat.) | | | | | |  | | |  | | | |  | | | |  | | | | |  |  |
| Managerial and tech. | 0.934  (0.65-1.34) | | 0.977  (0.72-1.33) | | | 0.812  (0.69-0.95) | | | 0.789  (0.64-0.97) | | | | 1.02  (0.8-1.3) | | | | 0.873  (0.74-1.02) | | | | |  |  |
| Skilled (non-manual) | 0.95  (0.7-1.28) | | 1.08  (0.83-1.4) | | | 0.874  (0.77-1) | | | 0.834  (0.7-1) | | | | 1.052 (0.86-1.29) | | | | 0.808  (0.71-0.92) | | | | |  |  |
| Skilled (manual) | 1.032  (0.74-1.45) | | 1.152  (0.87-1.53) | | | 0.875  (0.76-1.01) | | | 0.676  (0.56-0.82) | | | | 1.026 (0.83-1.28) | | | | 0.804  (0.7-0.93) | | | | |  |  |
| Partly-skilled | 0.936  (0.69-1.27) | | 0.985  (0.77-1.26) | | | 0.74  (0.65-0.84) | | | 0.645  (0.54-0.77) | | | | 0.89  (0.73-1.09) | | | | 0.89  (0.78-1.02) | | | | |  |  |
| Unskilled occupat. | 0.732  (0.52-1.02) | | 0.966  (0.73-1.29) | | | 0.713  (0.61-0.84) | | | 0.601  (0.49-0.73) | | | | 0.767 (0.62-0.96) | | | | 0.849  (0.73-0.99) | | | | |  |  |
